# Coordinated reset stimulation of plastic neural networks with spatially dependent synaptic connections

**DOI:** 10.3389/fnetp.2024.1351815

**Published:** 2024-05-28

**Authors:** Justus A. Kromer, Peter A. Tass

**Affiliations:** Department of Neurosurgery, Stanford University, Stanford, CA, United States

**Keywords:** coordinated reset stimulation, spatial neural networks, synchronization, spike-timing-dependent plasticity, networks of spiking neurons, desynchronization

## Abstract

**Background:**

Abnormal neuronal synchrony is associated with several neurological disorders, including Parkinson’s disease (PD), essential tremor, dystonia, and epilepsy. Coordinated reset (CR) stimulation was developed computationally to counteract abnormal neuronal synchrony. During CR stimulation, phase-shifted stimuli are delivered to multiple stimulation sites. Computational studies in plastic neural networks reported that CR stimulation drove the networks into an attractor of a stable desynchronized state by down-regulating synaptic connections, which led to long-lasting desynchronization effects that outlasted stimulation. Later, corresponding long-lasting desynchronization and therapeutic effects were found in animal models of PD and PD patients. To date, it is unclear how spatially dependent synaptic connections, as typically observed in the brain, shape CR-induced synaptic downregulation and long-lasting effects.

**Methods:**

We performed numerical simulations of networks of leaky integrate-and-fire neurons with spike-timing-dependent plasticity and spatially dependent synaptic connections to study and further improve acute and long-term responses to CR stimulation.

**Results:**

The characteristic length scale of synaptic connections relative to the distance between stimulation sites plays a key role in CR parameter adjustment. In networks with short synaptic length scales, a substantial synaptic downregulation can be achieved by selecting appropriate stimulus-related parameters, such as the stimulus amplitude and shape, regardless of the employed spatiotemporal pattern of stimulus deliveries. Complex stimulus shapes can induce local connectivity patterns in the vicinity of the stimulation sites. In contrast, in networks with longer synaptic length scales, the spatiotemporal sequence of stimulus deliveries is of major importance for synaptic downregulation. In particular, rapid shuffling of the stimulus sequence is advantageous for synaptic downregulation.

**Conclusion:**

Our results suggest that CR stimulation parameters can be adjusted to synaptic connectivity to further improve the long-lasting effects. Furthermore, shuffling of CR sequences is advantageous for long-lasting desynchronization effects. Our work provides important hypotheses on CR parameter selection for future preclinical and clinical studies.

## 1 Introduction

The human body consists of various interacting physiological systems forming a complex network in which stimulation of one system can have long-ranging effects on others (Bashan et al., 2012). To develop novel treatments, researchers study how the complex interactions between physiological systems and the various signaling pathways lead to the emergence of pathological and physiological states (Bartsch et al., 2015; Ivanov et al., 2016) and how transitions between these states can be induced, e.g., by stimulation.

Parkinson’s disease is accompanied by synaptic reorganization (Fan et al., 2012; Miguelez et al., 2012; Chu et al., 2017; Pamukcu et al., 2020; Madadi Asl et al., 2022) and changes of neuronal activity (Hammond et al., 2007; Neumann et al., 2016; Vinding et al., 2024) in several brain areas. The severity of motor symptoms, such as bradykinesia and rigidity, correlates with excessive neuronal synchrony (see, e.g., Hammond et al., 2007; Neumann et al., 2016). Brain stimulation has been found to alleviate symptoms, which goes along with a reduction of neuronal synchrony (Hammond et al., 2007). These observations motivated a model of a multistable complex network of PD-related brain areas (Tass and Majtanik, 2006; Tass and Hauptmann, 2007). In this model, pathological states characterized by excessive neuronal synchrony and the presence of symptoms (Hammond et al., 2007) and with reorganized synaptic connectivity (Mallet et al., 2019; Madadi Asl et al., 2022) coexist with healthy states characterized by reduced neuronal synchrony and less severe symptoms or even the absence thereof (Tass and Hauptmann, 2007).

High-frequency (HF) deep brain stimulation (DBS) is an established treatment for medically refractory PD (Krack et al., 2003). However, symptoms return shortly after cessation of stimulation (Temperli et al., 2003). Applying the interpretation of coexisting physiological and pathological states suggests that while HF DBS induces acute therapeutic effects during stimulation, it may not drive the network into the attractor of a stable, healthy state where the network remains after stimulation ceases.

CR stimulation is a multisite stimulation technique computationally developed to specifically counteract pathological neuronal synchrony (Tass, 2003a; Tass, 2003b). During CR stimulation, a spatiotemporal pattern of phase-shifted stimuli is delivered to multiple stimulation sites to target segregated neuronal subpopulations. Originally, CR stimulation was developed to desynchronize neuronal networks with fixed synaptic connections (Tass, 2003a; Tass, 2003b). Later, computational studies of CR stimulation in plastic networks revealed that stimulation not only reduced synchronization during stimulation but also caused downregulated synaptic connections, which drove the network into the attractor of a stable desynchronized state such that desynchronization effects persisted even after cessation of stimulation (Tass and Majtanik, 2006). Later preclinical studies in animal models for PD (Tass et al., 2012; Wang et al., 2016; Bore et al., 2022; Wang et al., 2022) and PD patients (Adamchic et al., 2014; Pfeifer et al., 2021) reported corresponding therapeutic effects that outlasted stimulation for several days to weeks. In contrast to HF DBS, this suggests that CR stimulation indeed drove the network into the attractor of a stable, healthy state, characterized by desynchronized neuronal activity, such that desynchronization effects persisted after cessation of stimulation.

Based on the computational results, the downregulation of (pathological, see Hauptmann and Tass, 2010) synaptic connectivity was key for inducing long-lasting desynchronization effects (Tass and Majtanik, 2006). Since then, several computational studies analyzed the role of different CR parameters for inducing long-lasting effects in plastic networks of phase oscillators (Tass and Majtanik, 2006), conductance-based neuron models (Popovych and Tass, 2012; Ebert et al., 2014; Lourens et al., 2015; Manos et al., 2018), and leaky integrate-and-fire (LIF) neurons (Kromer et al., 2020). These studies pointed out that the spatiotemporal pattern of stimulus deliveries, characterized by the stimulation frequency and the timing and the locations of stimulation site activations, has a strong impact on the CR-induced synaptic dynamics (Kromer et al., 2020; Khaledi-Nasab et al., 2021; Khaledi-Nasab et al., 2022). For CR stimulation, stimuli are delivered in cycles such that each stimulation site receives exactly one stimulus per cycle. The sequence at which sites receive stimuli is called CR sequence. In preclinical and clinical studies on CR, the CR sequence is typically shuffled after each cycle, which will be referred to as shuffled CR below (Tass et al., 2012; Adamchic et al., 2014; Wang et al., 2022). A recent computational and theoretical study also showed that the stimulus shape, characterized by the number of pulses per stimulus burst and the intraburst frequency, has a strong impact on the CR-induced synaptic dynamics (Kromer and Tass, 2022). Beyond the scope of CR stimulation other computational studies analyzed how the pattern of stimulus deliveries can shape plastic synaptic connections (see for instance, Madadi Asl et al., 2023; Anil et al., 2023), how synaptic reshaping might interact with heterogeneous intrinsic neuronal dynamics (Pariz et al., 2023), how spatially dependent connections impact synchronization (Berner and Yanchuk, 2021), and how stimulation-induced synaptic reshaping can lead to lasting effects on neuronal synchrony (Schmalz and Kumar 2019). However, none of these studies analyzed how different spatial patterns of synaptic connectivity might affect the CR-induced synaptic weight dynamics and how such spatial patterns can be harnessed for improved long-lasting effects of CR stimulation. The latter is an important question, as typical target brain regions, for instance, the subthalamic nucleus (STN) for CR DBS in PD, present complex patterns of synaptic connectivity (Emmi et al., 2020). Recently, a brief computational study reported that long-lasting effects of CR in spatially inhomogeneous networks might depend on the selected CR sequence and suggested that rapid shuffling of the CR sequence might be advantageous for long-lasting effects (Kromer and Tass, 2024), indicating that CR might have different effects depending on the synaptic network structure in the target brain area.

The present paper studies CR stimulation in plastic networks with spatially dependent synaptic connections. The network dynamics is modeled by LIF neurons with spike-timing-dependent plasticity (STDP) (Kromer and Tass, 2020). The network parameters are adjusted such that a strongly connected synchronized state and a weakly connected desynchronized state coexist. This was motivated by the presence of pathological states with excessive neuronal synchrony (Hammond et al., 2007) and healthy states characterized by the absence of excessive neuronal synchrony in PD (Tass and Majtanik, 2006). In contrast to previous studies, we vary the spatial structure of synaptic connections and derive stimulation strategies for different network structures. Specifically, we introduce a characteristic length scale of synaptic connections, *s*, that controls the distance dependence of the connection probability between two neurons. We assumed a decay of the connection probability with the distance between neurons, based on the analysis of synaptic connectivity in several brain regions, including the cortex (Hellwig, 2000), the striatum (Humphries et al., 2010), and as previously assumed in a detailed computational model of the STN (Ebert et al., 2014).

Based on our computational results, we derive the hypothesis that local connections dominate networks with short synaptic length scales and that selecting an appropriate stimulus shape has a significant impact on synaptic downregulation regardless of the spatiotemporal pattern of stimulus deliveries. In contrast, networks with long synaptic connections possess mainly connections between neurons close to different stimulation sites, and CR-induced synaptic downregulation strongly depends on the spatiotemporal pattern of stimulus deliveries.

Our paper is organized as follows. First, we present the neuronal network model and show that different stable states coexist for a wide range of synaptic length scales. In the results section, we present our simulation results on CR stimulation of networks of LIF neurons with STDP and with different synaptic length scales and analyze the stimulation-induced network structure. Then, we derive efficient stimulation strategies for such networks and show how the spatiotemporal stimulus pattern affects the dynamics of synaptic connections. Finally, we discuss our results.

## 2 Model and methods

### 2.1 Network of leaky integrate-and-fire neurons

To simulate the effect of CR stimulation on plastic neural networks, we performed simulations of networks of excitatory LIF neurons with STDP. The model equations were taken from previous studies (Kromer et al., 2020; Kromer and Tass, 2020). Here, we extend the model by considering networks with different synaptic length scales as described below.

Individual neurons were modeled as LIF neurons. The *i*th neuron’s membrane potential, *V*_*i*_(*t*), obeyed the dynamics.

$$C_{i}\frac{dV_{i}}{dt}=g_{\text{leak}}\left(V_{\text{rest}}-V_{i}\right)+g_{\text{syn},i}\left(t\right)\left(V_{\text{syn}}-V_{i}\right)+I_{\text{stim},i}\left(t\right)+I_{\text{noise},i}\left(t\right).$$ (1)

Here, *C*_*i*_ is the membrane capacitance and the terms on the right-hand side model the leak current, with leakage conductance *g*_leak_ and resting potential *V*_rest_; the excitatory synaptic input current, with synaptic conductance *g*_syn,*i*_(*t*) and reversal potential *V*_syn_; the stimulation current *I*_stim,*i*_(*t*); and the noisy input current *I*_noise,*i*_(*t*), modelling input from other brain regions. Action potentials (spikes) were not modelled directly. Instead neuron *i* was assumed to exhibit a spike whenever its membrane potential crossed the threshold potential *V*_th,*i*_(*t*) from below. *V*_th,*i*_(*t*) obeyed the dynamics

$$\tau_{\text{th}}\frac{dV_{\text{th},i}}{dt}=-\left(V_{\text{th},i}-V_{\text{th,rest}}\right).$$ (2)

After threshold crossing, we implemented a rectangular voltage spike by setting *V*_*i*_(*t*) to *V*_spike_ for a duration of *τ*_spike_. Then, we performed an instantaneous reset of the threshold potential and the membrane potential: *V*_th,*i*_(*t*) → *V*_th,spike_ and *V*_*i*_(*t*) → *V*_reset_.

The synaptic conductances *g*_syn,*i*_(*t*) were increased instantaneously whenever a presynaptic spike arrived, and decayed exponentially in between incoming presynaptic spikes:

$$\tau_{\text{syn}}\frac{dg_{\text{syn},i}}{dt}=-g_{\text{syn},i}+\kappa \frac{\tau_{\text{syn}}}{N}\underset{j\in G_{i}}{\sum }w_{j\to i}\left(t\right)\underset{l\in Y_{j}}{\sum }\delta \left(t-t_{l}^{j}-t_{\text{d}}\right).$$ (3)

*τ*_syn_ is the synaptic timescale, $t_{l}^{j}$ the timing of the *l*th spike of neuron *j*, *Y*_*j*_ the set of spikes of neuron *j*, and *t*_d_ the synaptic (axonal) transmission delay. The first sum runs over the set of all presynaptic neurons *G*_*i*_ of neuron *i*. *κ* scales the maximum synaptic coupling strength. Additionally, the strength of individual synapses was scaled by the time-dependent synaptic weights *w*_*j*→*i*_(*t*), with *j* being the index of the presynaptic neuron and *i* the index of the postsynaptic neuron.

To model noisy input from other brain areas, we delivered independent Poisson spike trains with mean firing rate *f*_noise_ to each neuron. These spike trains were fed into the neurons through excitatory synapses. The resulting input current to neuron *i* is given by

$$I_{\text{noise},i}\left(t\right)=g_{\text{noise,i}}\left(t\right)\left(V_{\text{syn}}-V_{i}\right).$$ (4)

*g*_noise,i_(*t*) is the noise conductance given by

$$\tau_{\text{syn}}\frac{dg_{\text{noise},i}}{dt}=-g_{\text{noise,i}}+\frac{\kappa_{\text{noise}}\tau_{\text{syn}}}{N}\underset{k\in U_{i}}{\sum }\delta \left(t_{k}-t\right).$$ (5)

*κ*_noise_ sets the noise intensity and *t*_*k*_ is the timing of the *k*th spike of the Poisson spike train that was fed into neuron *i*, and *U*_*i*_ is the set of all spikes in this spike train.

All parameters were taken from Kromer and Tass (2020): *g*_leak_ = 0.02 mS/cm^2^, *V*_rest_ = −38 mV, *V*_reset_ = −67 mV, *V*_th,spike_ = 0 mV, *V*_th,rest_ = −40 mV, *τ*_th_ = 5 ms, *V*_syn_ = 0 mV, *τ*_syn_ = 1 ms, *t*_d_ = 3 ms, *κ* = 8 mS/cm^2^, *κ*_noise_ = 0.026 mS/cm^2^, and *f*_noise_ = 20 Hz. The membrane capacitances *C*_*i*_ were Gaussian distributed ( $N\left(\mu_{\text{C}},\sigma_{\text{C}}\right)$ , with mean value *μ*_C_ = 3 *μ*F/cm^2^ and standard deviation *σ*_C_ = 0.05*μ*_C_). This led to heterogeneity of the intrinsic dynamics of individual neurons. This parameter set was chosen such that the frequency and the range of membrane potential oscillations matched recordings of periodically spiking STN neurons in brain slices of healthy rats (Bevan and Wilson, 1999). The STN is a major target region for HF DBS in PD (Krack et al., 2003) and target for CR DBS in Parkinsonian monkeys (Tass et al., 2012) and PD patients (Adamchic et al., 2014).

### 2.2 Networks with spatially dependent synaptic connections

To study CR stimulation in networks with spatially dependent synaptic connectivity, we considered networks of *N* = 1000 LIF neurons. The neurons were placed along the *x*-axis such that their center coordinates, *x*_*i*_, were uniformly distributed in the interval 0 ≤ *x*_*i*_ < *L*. This is a similar distribution as in previous computational studies (Kromer et al., 2020) and was motivated by the distribution of neurons along a DBS electrode. Synaptic connections were introduced randomly between the neurons such that 7% of all *N* ^2^ possible connections are implemented. This was motivated by earlier detailed studies on the effect of CR stimulation on neuronal activity in a detailed model of the STN (Ebert et al., 2014). We did not allow for autaptic connections.

**FIGURE 1:**
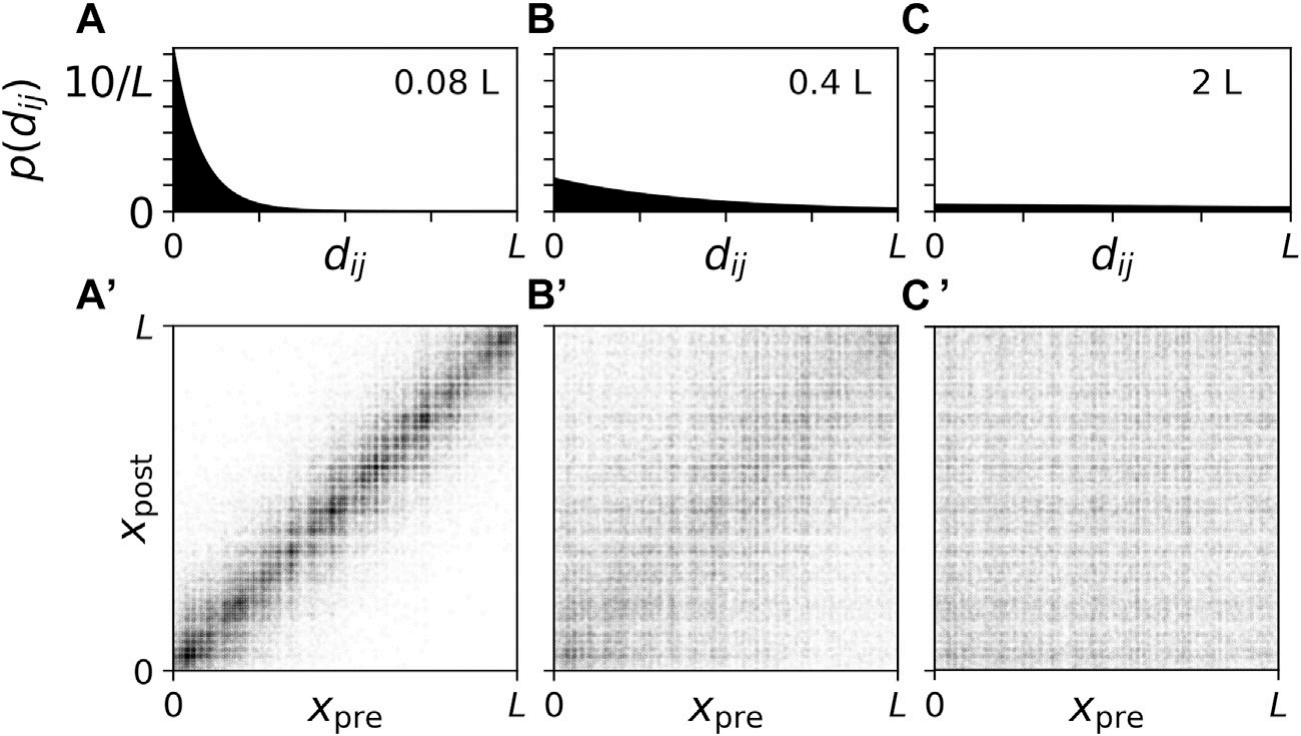
Connection probability and connectivity diagrams for network realizations with different synaptic length scales. **(A–C)**: Plots of the connection probability as a function of the distance, *d*_*ij*_, between two neurons. We normalized *p* (*d*_*ij*_) such that $\int_{0}^{\infty }du\;p\left(u\right)=1$ . Labels indicate synaptic length scales. **(A’–C’)** Corresponding connectivity diagrams. Black dots indicate a synaptic connection between a presynaptic neuron at location *x*_pre_ and a postsynaptic neuron at location *x*_post_. Panels show results for synaptic length scales *s* =0.08*L* **(A, A′)**, 0.4*L* **(B, B′)**, and 2.0*L* **(C, C′)**. Here, *L* is the system’s length scale. In units of the distance between adjacent stimulation sites, *d*, the synaptic lengths scales are *s* =0.32 d **(A)**, 1.6 d **(B)**, and 8.0 d **(C)** (see below).

The spatial dependence of the synaptic connections was implemented by considering a distance-dependent connection probability *p* (*d*_*ij*_) ∝ exp (−*d*_*ij*_/*s*) (Ebert et al., 2014). Here, *d*_*ij*_ = |*x*_*j*_ − *x*_*i*_| is the Euclidian distance between the presynaptic neuron *i* and the postsynaptic neuron *j*. Periodic boundary conditions were not considered. *s* is the synaptic length scale and will be varied throughout the present paper. Network realizations for the three values of *s* that were considered are visualized in Figure 1.

For *s* ≪ *L* the network of synaptic connections is dominated by local connections whereas *s* ≫ *L* leads to rather homogeneous synaptic connectivity that becomes independent of the distance between neurons in the limit of long synaptic length scales *s*/*L* → *∞*. In the present paper, we studied how CR stimulation reshapes the synaptic connectivity for the three synaptic length scales in Figure 1 which are representative of cases where the network is dominated by local connections (*s* = 0.08 L, Figures 1A, A’), the network shows close-to-homogeneous synaptic connectivity (*s* = 2 L, Figures 1C, C’), and a case in between these two extreme cases (*s* = 0.4 L, Figures 1B, B’).

For comparison, we discuss values of *L* and *s* used in previous studies modelling the STN. In Kromer et al. (2020), *L* = 5.0 mm was used, which was motivated by the length of a short axis of an ellipsoidal volume approximation of the STN based on experimental measurements of STN tissue volume (Lévesque and Parent, 2005; Ebert et al., 2014). Magnetic resonance imaging (MRI) studies reported variations of STN dimensions among PD patients with measured mean lengths of 5.9, 3.7, and 5.0 mm along the anteroposterior, mediolateral, and dorsoventral axes, respectively; however, it was noted that MRI yields smaller estimates of STN dimensions than other techniques (Richter et al., 2004). In a detailed computational study, *s* = 0.5 mm was used to reproduce experimentally measured mean connection lengths (Afsharpour, 1985; Hellwig, 2000; Ebert et al., 2014). Together, these values for *L* and *s* would correspond to a synaptic length scale of *s* ≈ 0.1*L*, close to the values used in Figures 1A, A’. However, note that the STN is part of the basal ganglia, and its activity is shaped by input from other nuclei (Emmi et al., 2020), making a comparison difficult.

### 2.3 Spike-timing-dependent plasticity

To model synaptic plasticity, we employed a nearest neighbor STDP scheme (Morrison et al., 2008). The synaptic weights *w*_*i*→*j*_ were updated whenever a presynaptic or a postsynaptic spike arrived at the synapse. Respective arrival times are given by *t*_pre_ + *t*_*d*_ for presynaptic and *t*_post_ for postsynaptic spikes. *t*_pre_ and *t*_post_ are the spike times of the presynaptic and postsynaptic neurons, respectively. At the arrival times, instantaneous synaptic weight updates were performed according to *w*_*i*→*j*_ → *w*_*i*→*j*_ + *W* (Δ*t*) with the time lag Δ*t* = *t*_post_ − *t*_pre_ − *t*_*d*_ and the STDP function (Song et al., 2000)

$$W\left(\Delta t\right)=\eta \;\left\{\begin{array}{cc} \exp\left(-\frac{\Delta t}{\tau_{+}}\right),\; & \;\;\Delta t>0,\\ 0,\; & \;\;\Delta t=0,\\ -\frac{\beta }{\tau_{\text{R}}}\exp\left(-\frac{\left|\Delta t\right|}{\tau_{-}}\right),\; & \;\;\Delta t<0. \end{array}\right.$$ (6)

*η* ≪ 1 scales the weight update per spike arrival. 1/*η* sets the time scale on which STDP affects the synaptic weights. The parameters *τ*_+_ and *τ*_−_ = *τ*_R_ *τ*_+_ determine the shape of the STDP function and are typically in the range of tens of milliseconds (Bi and Poo, 1998). *β* scales the ratio of overall long-term depression to long-term potentiation, i.e., the integrals over the time lags for which *W*(Δ*t*) < 0 $\left(\left|\int_{-\infty }^{0}dt\;W\left(t\right)\right|\right)$ and for which *W* (Δ*t*) > 0 $\left(\left|\int_{0}^{\infty }dt\;W\left(t\right)\right|\right)$ , respectively. STDP is typically considered to be either balanced (*β* ≈ 1), depression dominated (*β* > 1), or potentiation dominated (*β* < 1). In the simulations, we used hard bounds by clipping the synaptic weights after each update to ensure that *w*_*i*→*j*_(*t*) ∈ [0, 1].

**FIGURE 2:**
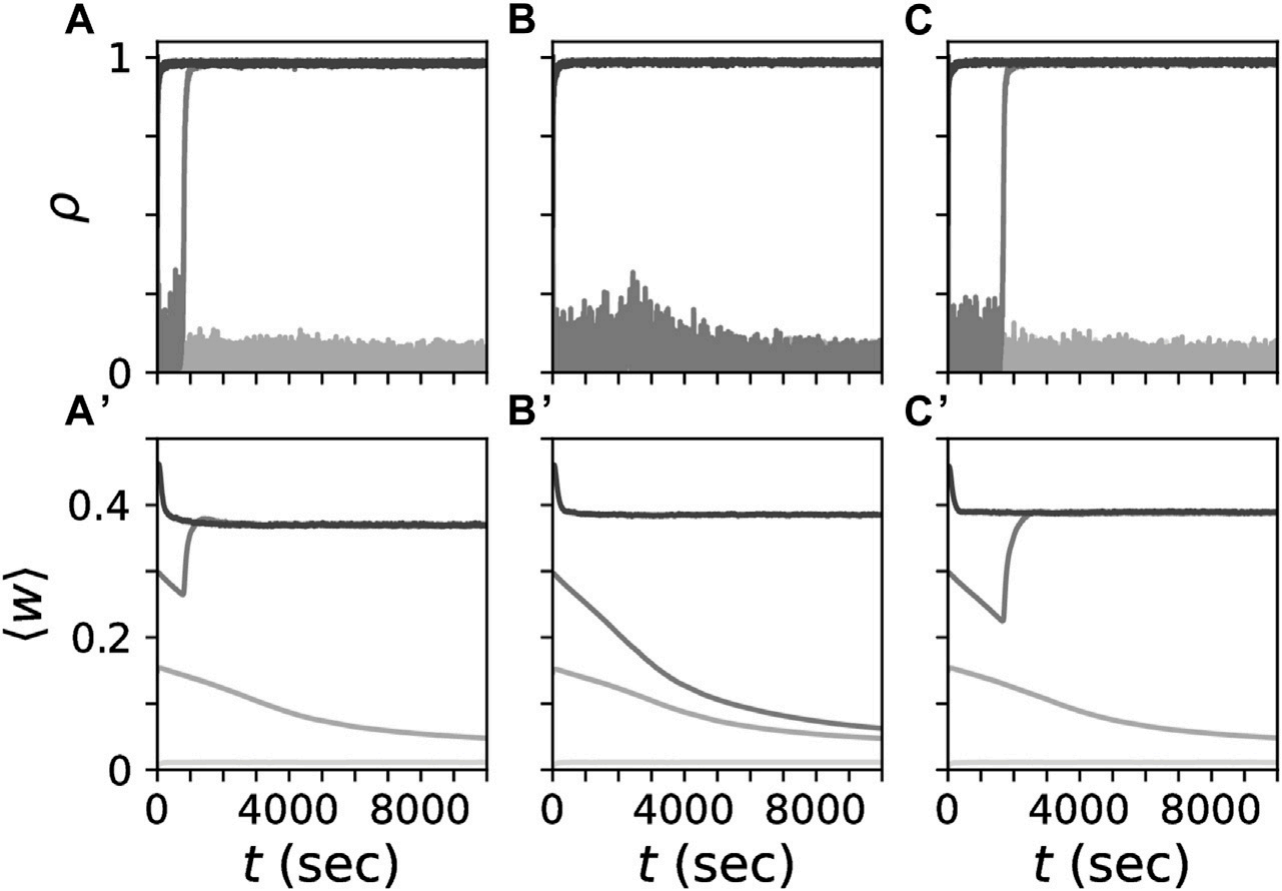
Coexistence of strongly connected synchronized and weakly connected desynchronized states for different synaptic length scales. Simulated traces of the Kuramoto order parameter *ρ*, Eq. 7, **(A–C)** for networks with different synaptic length scales, *s* (columns). Panels in the bottom row show corresponding traces of the mean synaptic weight, ⟨*w*⟩ obtained by averaging the weights of all synapses in the network. Gray tones mark trajectories for different initial mean synaptic weights. At *t* = 0 individual synaptic weights were randomly set to either zero or one such that a given mean synaptic weight was realized. Synaptic length scales were the same as in Figure 1, i.e., *s* = 0.08*L* **(A,A** **′** **)**, *s* = 0.4*L* **(B,B** **′** **)**, *s* =2*L* **(C,C** **′** **)**.

Throughout the present paper, we used the parameters *β* = 1.4, *τ*_+_ = 10 ms, *τ*_R_ = 4 (Kromer et al., 2020; Kromer and Tass, 2020). These parameters ensured that desynchronized and synchronized states coexisted for all considered synaptic length scales (Figure 2). We set *η* = 0.01 which yielded slow changes of the synaptic weights relative to the interspike intervals.

### 2.4 Degree of in-phase synchronization

The degree of in-phase synchronization was quantified by evaluating the Kuramoto order parameter (Kuramoto, 1984)

$$\rho \left(t\right)≔\left|\frac{1}{T}\overset{N}{\underset{i=1}{\sum }}e^{2\pi I\phi_{i}\left(t\right)}\right|.$$ (7)

*ϕ*_*i*_(*t*) is a phase function that attains subsequent integer values at the timings of subsequent spikes of neuron *i* and increases linearly in between subsequent spikes (Rosenblum et al., 2001). I is the imaginary unit. *ρ*(*t*) ≈ 1 indicates in-phase synchronization and *ρ*(*t*) ≈ 0 the absence of in-phase synchronization.

### 2.5 Coordinated reset stimulation

CR stimulation was delivered to four stimulation sites (Tass, 2003b). The sites were placed at locations *x*_*K*_ = *L*/8, 3*L*/8, 5*L*/8, and 7*L*/8, respectively, such that the distance between two adjacent sites was *d* = *L*/4.

**FIGURE 3:**
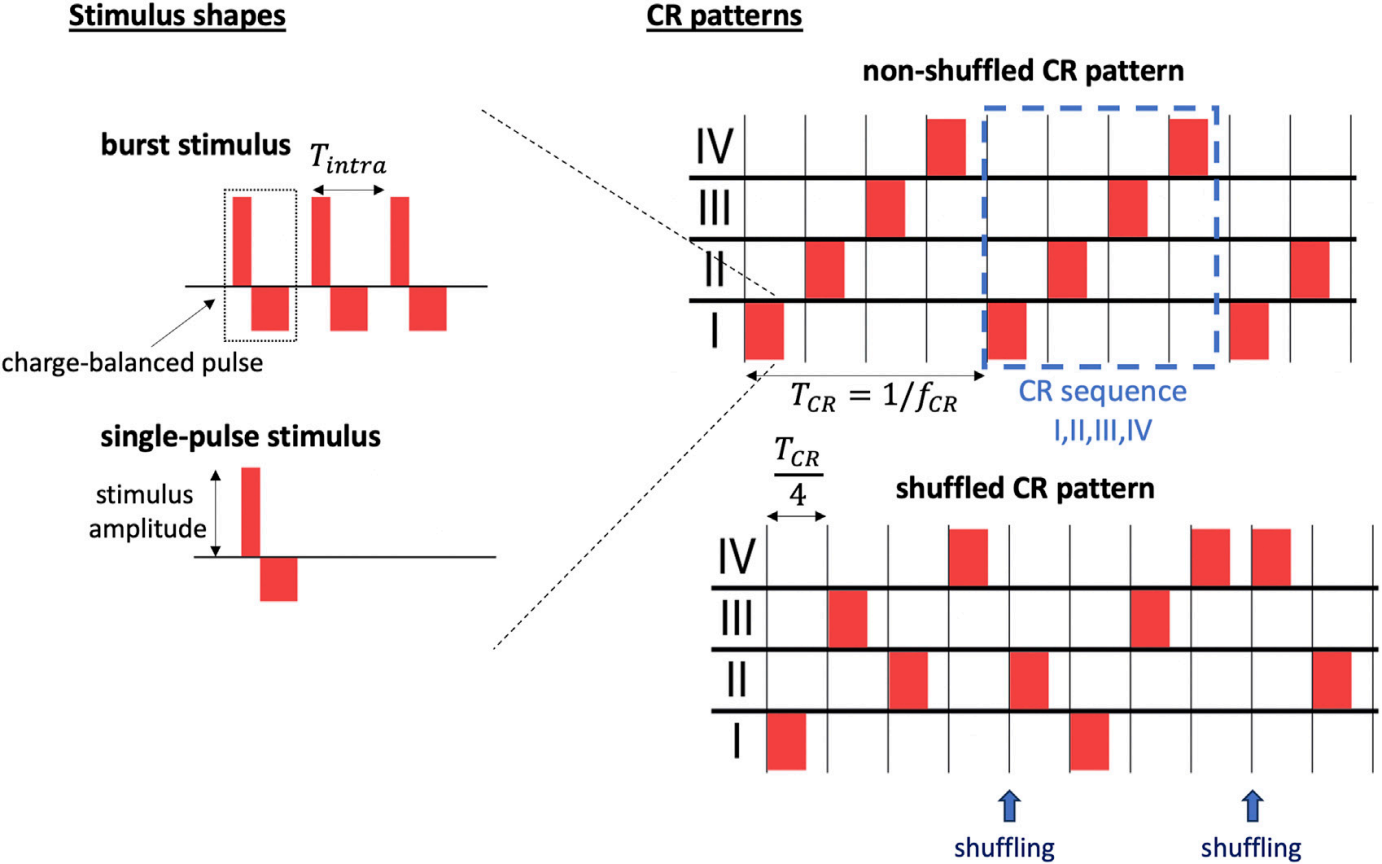
Illustration of stimuli and CR pattern-related parameters. We distinguish between individual stimuli and the spatio-temporal pattern of stimulus deliveries (CR pattern). Individual stimuli are characterized by the stimulus shape (left). Two different stimulus shapes are considered throughout the present paper: burst stimuli consisting of three charge-balanced pulses separated by *T*_intra_ = 1/*f*_intra_ (top left) and single-pulse stimuli (bottom left). Stimulus-related parameters include the stimulus amplitude, the number of pulses per stimulus, the intraburst frequency, *f*_intra_, and the waveform during individual charge-balanced pulses, here characterized by the widths of the excitatory and the inhibitory rectangular pulses (left). Several CR patterns are used throughout the present paper (right). In the right panels, red rectangles mark individual stimuli. We distinguish between non-shuffled CR patterns in which the same CR sequence is repeated for the entire stimulation period (top right) and shuffled CR patterns in which a new CR sequence of stimulus deliveries to the stimulation sites (Roman numerals) is generated after each cycle period, *T*_CR_, by shuffling the *M* = 4 Roman numerals, referring to individual sites, and randomly drawing *M* Roman numerals without replacement (bottom right). The shuffled CR pattern shown in the bottom right consists of the CR sequences “I,III,II,IV”, and before the first shuffling, and “II,I,III,IV”, after the first shuffling. After the second shuffling, only the first half of a CR sequence is shown with stimulus deliveries to sites IV and II.

Stimulation was delivered in cycles of four stimuli *T*_CR_/4, such that each site received exactly one stimulus per cycle. The cycle period *T*_CR_ is given by the inverse CR frequency *T*_CR_ = 1/*f*_CR_. Hence, individual sites received stimuli at an average inter-stimulus interval of *T*_CR_. Following, we refer to the sequence of stimulus deliveries during a single CR cycle as *CR sequence* and to the overall spatio-temporal pattern of stimulus deliveries as *CR pattern* (Figure 3). We considered two qualitatively different CR patterns: non-shuffled CR and shuffled CR. During non-shuffled CR, one of the 4! possible CR sequences is selected and delivered for the entire stimulation period. In contrast, during shuffled CR, a new CR sequence is randomly selected at the beginning of each CR cycle, i.e., every *T*_CR_ (Figure 3). Shuffled CR has been used in preclinical (Tass et al., 2012) and clinical studies (Adamchic et al., 2014) on PD.

Individual stimuli were modelled as a sequence of charge-balanced pulses. In neuroscience and medicine, electrical stimuli are typically charge-balanced to avoid tissue damage (Harnack et al., 2004). Each charge-balanced pulse consisted of two rectangular current pulses: an excitatory one and an inhibitory one. The excitatory one had a pulse duration of *ν*_e_ = 0.4 ms and an amplitude of $A_{\text{e}}=A\Delta V\mu_{\text{C}}/\nu_{\text{e}}$ . It was immediately followed by the inhibitory one, which had a duration of *ν*_i_ = 0.8 ms and an amplitude of $A_{\text{i}}=-A_{\text{e}}\nu_{\text{e}}/\nu_{\text{i}}$ (Figure 3). Δ*V* = *V*_th,spike_ − *V*_reset_ is the maximum voltage range in the subthreshold voltage region of a single LIF neuron in the absence of stimulation and *A* the dimensionless stimulation strength. *A* = 1 marks stimuli for which the excitatory pulse resulted in an approximate elevation of the membrane potential of Δ*V*. Thus, individual stimuli typically elicited a spiking responses.

We employed a spatial stimulus profile to account for the fact that neurons that are further away from a stimulation site, experience weaker stimuli than those close to the site (Richardson et al., 2003). This effectively reduced the stimulus amplitude for neurons at a finite distance |*x*_*i*_ − *x*_*K*_| from a site located at *x*_*K*_. Specifically, an LIF neuron with center coordinated *x*_*i*_ experienced stimuli with a reduced amplitude *A*(*x*_*i*_, *x*_*K*_) from a site at *x*_*K*_ (Lysyansky et al., 2013)

$$A\left(x_{i},x_{K}\right)=A_{stim}{\left(1+{\left(\frac{x_{i}-x_{K}}{\sigma }\right)}^{2}\right)}^{-1}.$$ (8)

*A*(*x*_*i*_, *x*_*K*_) is the dimensionless stimulus amplitude experienced by a neuron at location *x*_*i*_ for a stimulus delivered to the stimulation site at location *x*_*K*_. *σ* scales the width of the stimulus profile and was set to *σ* = *d*/4*π* throughout the present paper. Thus, during a stimulus delivery to a site at *x*_*K*_ a neuron at location *x*_*i*_ received an excitatory stimulation current, *I*_stim,*i*_(*t*), (Eq. 1), with amplitude *A*(*x*_*i*_, *x*_*K*_)Δ*Vμ*_C_/*ν*_e_, during the excitatory rectangular pulses and a current with amplitude −*A*(*x*_*i*_, *x*_*K*_)Δ*Vμ*_C_/*ν*_i_ during the inhibitory pulses.

Below, we study the impact of the *stimulus shape*, referring to the time course of *I*_stim,*i*_(*t*) after stimulus onset, on the outcome of CR stimulation. To this end, we deliver single-pulse stimuli, consisting of one charge-balanced pulse, i.e., one excitatory rectangular pulse followed by an inhibitory one as described above, and burst stimuli consisting of multiple such charge-balanced pulses (Figure 3). Within a burst stimulus, the time lag between subsequent charge-balanced pulses was controlled by the intraburst frequency, *f*_intra_, specifying the inverse time between the onset of two subsequent charge-balanced pulses within a burst.

### 2.6 Analytical approximation of relative number of synaptic connections between neuronal subpopulations affected by two stimulation sites

In the results section, we present approximations for the mean synaptic weight based on the relative numbers of synaptic connections *b*_*xy*_≔*B*_*xy*_/*∑*_*xy*_ *B*_*xy*_ between a presynaptic neuronal subpopulation *x* and a postsynaptic neuronal subpopulation *y*. In particular, we consider neuronal subpoplations that are the closest to one of the stimulation sites. Here, *B*_*xy*_ is the total number of connections between the two neuronal subpopulations. The neuronal subpopulation that is the closest to stimulation site *K* at location *x*_*K*_ = (2*K* − 1)*L*/8, *K* = 1, 2, 3, 4 incorporates all neurons with center coordinates *x*_*i*_ ∈ [(*K* − 1)*L*/4, *KL*/4).

Next, we consider the distribution *p*_Δ*K*_(*d*_*ij*_, *d*) of distances *d*_*ij*_ between postsynaptic neurons *j* that are the closest to site *K*_post_ and presynaptic neurons *i* that are the closest to stimulation site *K*_pre_ with Δ*K* = *K*_post_ − *K*_pre_. For a uniform distribution of neuron center coordinates in each subpopulation, we find.

$$p_{\Delta K}\left(d_{ij},d\right)=\frac{1}{d}\left\{\begin{array}{cc} \left(\frac{d_{ij}}{d}-\left(\Delta K-1\right)\right),\; & d\left(\Delta K-1\right)\leq d_{ij}<d\Delta K\\ \left(-\frac{d_{ij}}{d}+\left(\Delta K+1\right)\right),\; & d\Delta K\leq d_{ij}<d\left(\Delta K+1\right)\\ 0,\; & \text{otherwise} \end{array}\right..$$ (9)

For a large number of connections between individual subpopulations, the relative numbers of connections between subpopulations can by approximated by,

$$b_{xy}\approx \frac{\overset{\infty }{\underset{-\infty }{\int }}dx\;p_{y-x}\left(dx,d\right)e^{-\frac{\left|dx\right|}{s}}}{\overset{M}{\underset{x,y=1}{\sum }}\overset{\infty }{\underset{-\infty }{\int }}dx\;p_{y-x}\left(dx,d\right)e^{-\frac{\left|dx\right|}{s}}}.$$ (10)

Here, *M* = 4 is the total number of stimulation sites. This integral can be solved analytically, and the solution depends only on the ratio *l* = *d*/*s* of the distance between adjacent stimulation sites *d*, characterizing the characteristic length scale of the spatiotemporal stimulus pattern, and the synaptic length scale *s*. In particular, for *l* → 0 the result becomes independent of *y* and *x* and yield results for homogeneous networks, which have been studied in Kromer and Tass (2022).

### 2.7 Approximation of mean synaptic weight after CR stimulation in networks with spatially dependent synaptic connections

Below, we explore the effect of the stimulus shape and the CR pattern on the mean synaptic weight after CR stimulation in more detail and develop effective stimulation strategies for synaptic weight reduction in networks with spatially dependent synaptic connections. Recently, we derived theoretical approximations of the stimulation-induced dynamics of the mean synaptic weight for non-shuffled CR stimulation with rectangular stimulation profile in networks with spatially homogeneous synaptic connectivity and STDP (Kromer and Tass, 2022). Given an estimate of the average rate of synaptic weight change during stimulation, *J*_*x*→*y*_, of synapses between the presynaptic neuronal population *x* and postsynaptic neuronal population *y* and the mean synaptic weight of these synapses at a reference time *t*_0_, ⟨*w*_*x*→*y*_ (*t*_0_)⟩, their mean synaptic weight at a later time *t* > *t*_0_ can be approximated by Kromer and Tass (2022)

$$\left\langle w_{x\to y}\left(t\right)\right\rangle \approx {\left[\left\langle w_{x\to y}\left(t_{0}\right)\right\rangle +S\left(J_{x\to y},\left\langle w_{x\to y}\left(t_{0}\right)\right\rangle \right)t-t_{0}\right]}_{\text{clip},0,1}.$$ (11)

${\left[a\right]}_{\text{clip},0,1}=a$ for *a* ∈ [0, 1], 0 for *a* < 0 and 1 for *a* > 1 accounts for the hard bounds for individual synaptic weights. The function

$$S\left(J,w\right)t-t_{0}≔\left\{\begin{array}{cc} wJ\left(t-t_{0}\right),\; & J\leq 0\\ \left(1-w\right)J\left(t-t_{0}\right),\; & J>0 \end{array}\right.,$$ (12)

accounts for a linear increase of the weights of initially downregulated synapses and a linear decrease of weights of initially upregulated ones. Note that during the derivation of Eq. 11, it was assumed that a mean synaptic weight of *w*_*x*→*y*_(*t*_0_) corresponds to a fraction of *w*_*x*→*y*_(*t*_0_) synapses with synaptic weights one and a fraction of 1 − *w*_*x*→*y*_(*t*_0_) synapses with synaptic weights zero. This assumption was motivated by the observation that individual synaptic weights are close to the hard bounds in the stationary states (Song et al., 2000; Rubin et al., 2001).

From Eq. 11, an approximation of the mean synaptic weight was derived in Kromer and Tass (2022). To this end, the matrix *B* was introduced with its entries *B*_*xy*_ being the total numbers of synaptic connections with presynaptic neurons in neuronal subpopulation *x* and postsynaptic neurons in subpopulaton *y*. For a given matrix *B*, the overall mean synaptic weight ⟨*w*(*t*)⟩ can be approximated by

$$\left\langle w\left(t\right)\right\rangle \approx \frac{1}{\sum_{xy}B_{xy}}\underset{xy}{\sum }B_{xy}\left\langle w_{xy}\left(t\right)\right\rangle =\underset{xy}{\sum }b_{xy}\left\langle w_{xy}\left(t\right)\right\rangle .$$ (13)

The sums run over all possible combinations of presynaptic neuronal subpopulations *x* and postsynaptic subpopulations *y*. *b*_*xy*_≔*B*_*xy*_/*∑*_*xy*_ *B*_*xy*_ denotes the relative number of connections with presynaptic neuron in subpopulation *x* and postsynaptic neuron in subpopulation *y*.

In the previous section, we derived an analytical estimate for the relative number of connections *b*_*xy*_ for the networks of spatially dependent synaptic connections considered throughout the present paper for the case where a subpopulation incorporates the neurons that are closest to one of the stimulation sites (Eq. 10). Using Eq. 10 in Eq. 13, we calculated the approximate dynamics of the overall mean synaptic weight during non-shuffled CR stimulation with a given CR sequence and a given ratio *l* of the distance between adjacent stimulation sites *d* and the synaptic length scale *s*. In Figure 7, results from Eq. 10 are compared to estimates from networks generated by the algorithm, used in our simulations. Note that Eq. 10 also yields the relative portion of intra-population synapses as *b*_intra_ = *∑*_*x*_ *B*_*xx*_ and inter-population synapses *b*_inter_ = 1 − *b*_intra_, i.e., synaptic connections between presynaptic and postsynaptic neurons that are closest to the same and to different sites, respectively.

### 2.8 Details of numerical simulations and data analyses

Numerical integration of the network model of LIF neurons with STDP was performed using an Euler scheme with integration time step 0.1 ms. The python code used for the simulations and the generation of the figures is available on github.

**FIGURE 4:**
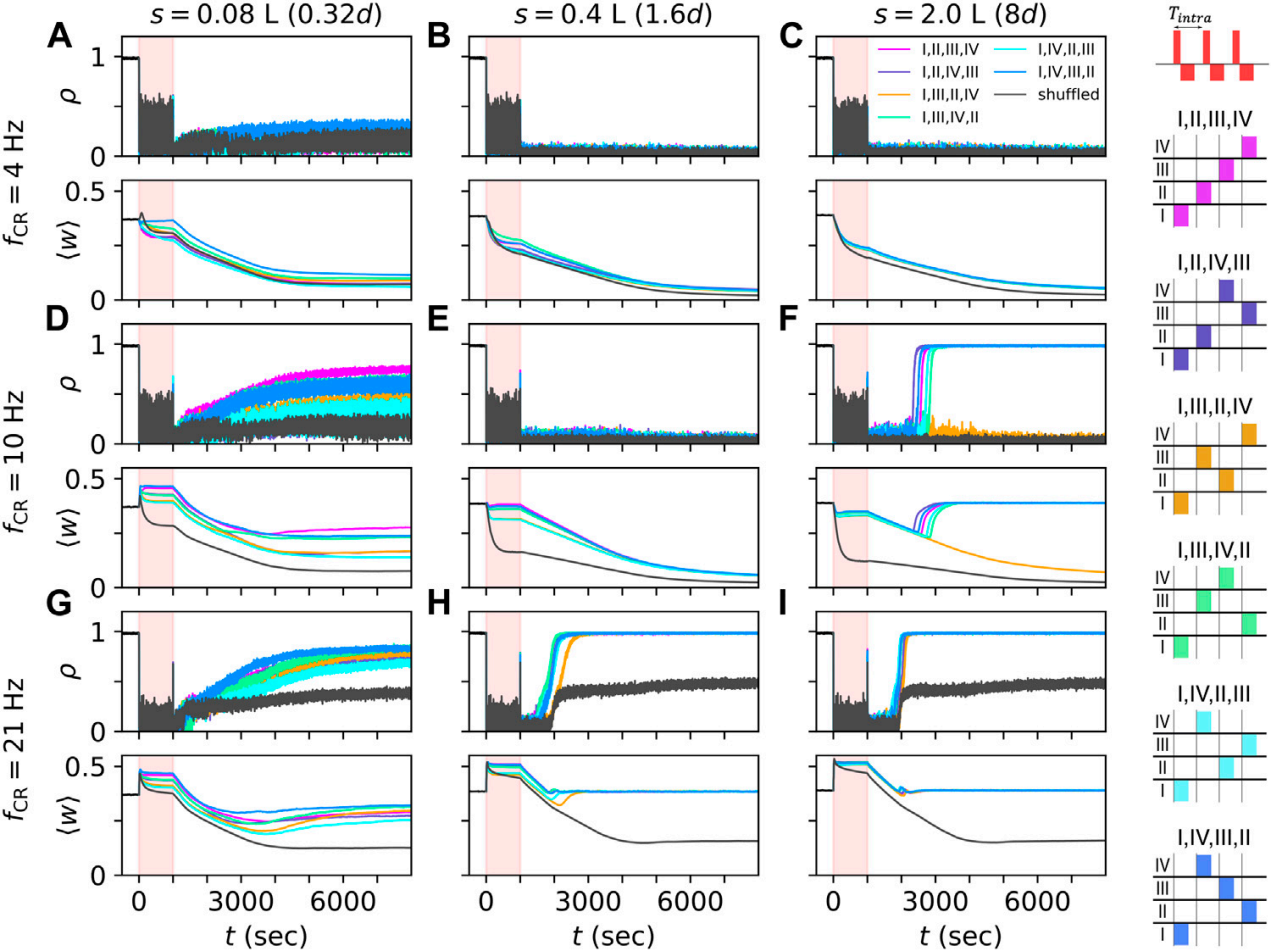
CR stimulation-induced dynamics depend on network structure. Simulated traces of the Kuramoto order parameter *ρ*, Eq. 7, for networks with different synaptic length scales. *s* = 0.08 L **(A,D,G)**, 0.4 L **(B,E,H)**, and 2.0 L **(C,F,I)**, and for different stimulation frequencies, *f*_CR_ = 4 Hz **(A–C)**, 10 Hz **(D–F)**, and 21 Hz **(G–I)**. Individual stimuli consisted of bursts of three stimulus pulses and an intraburst frequency of 130 Hz, the corresponding time between subsequent pulses within a burst, *T*_intra_, is 1/130 s (see schematic on the top right). Black traces show results for shuffled CR, often referred to as CR with rapidly varying sequence (Tass and Majtanik, 2006; Zeitler and Tass, 2015)). The stimulation period of 1000 s is marked in light red. Colored traces show results for non-shuffled CR with CR sequences “I,II,III,IV”,“I,II,IV,III”,“I,III,II,IV”,“I,III,IV,II”,“I,IV,II,III”, and “I,IV,III,II”. The corresponding sequences of stimulation site activations within a CR cycle are illustrated on the right-hand side. Parameters: *A*_stim_ = 2.5 and *σ* = *d*_s_/4 Π.

**FIGURE 5:**
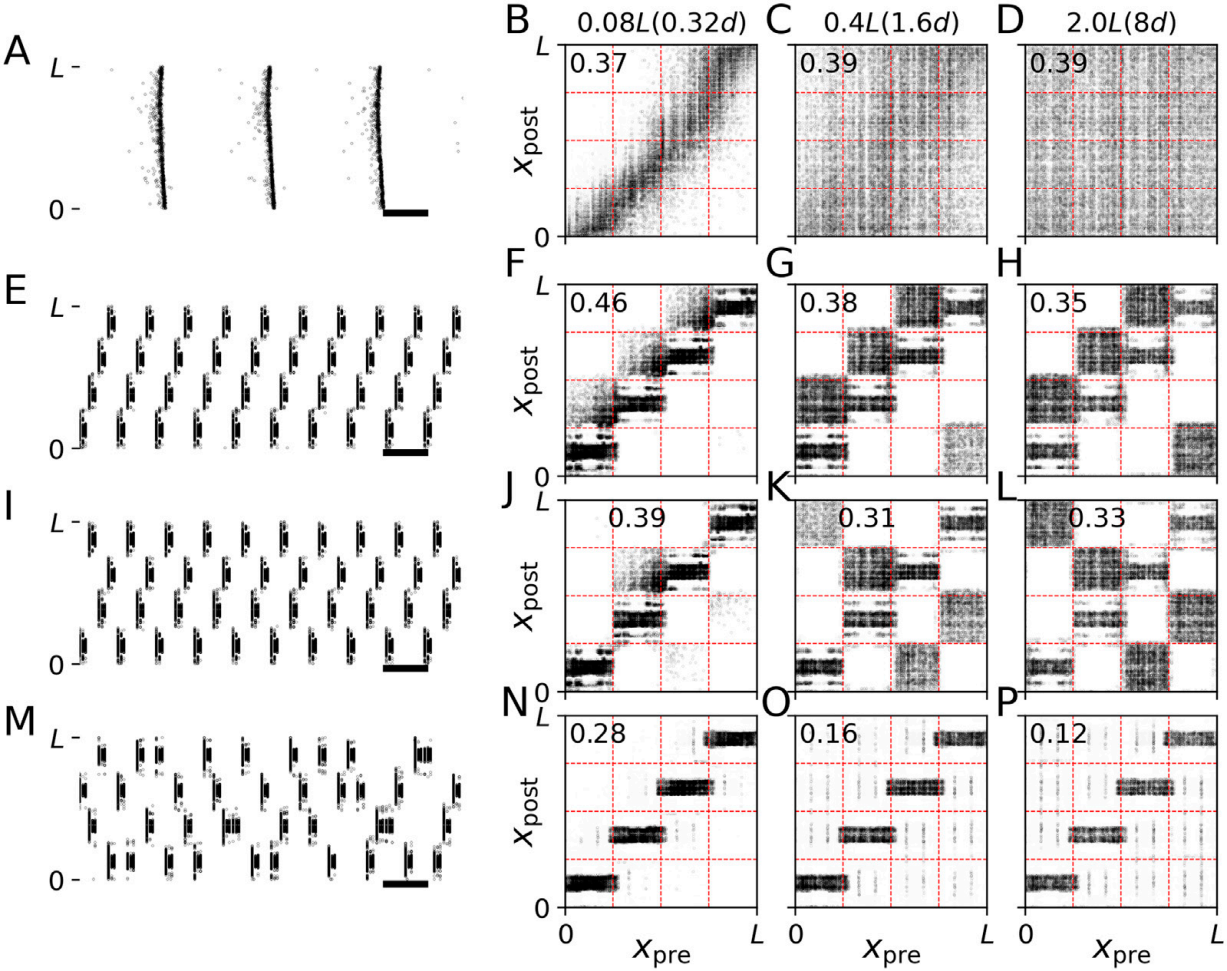
Synaptic pathways induced by CR stimulation with burst stimuli. Results of simulations of the LIF network model prior to stimulation **(A–D)**, and during stimulation with non-shuffled CR with CR sequences “I,II,III,IV” **(E–H)** and “I,IV,II,III” **(I–L)** and shuffled CR **(M–P)** are shown. Left panels show raster plots of neuronal spiking activity. Black horizontal bars mark a time interval of 100 ms. The other panels show connectivity diagrams with dark colors marking strong connections and light gray marking weak connections between presynaptic neurons at locations *x*_pre_ and postsynaptic neurons at locations *x*_post_. Different columns show results for networks with different synaptic length scales, *s* (see labels at the top of respective columns). In the brackets, we give synaptic length scales in units of the distance between adjacent stimulation sites, *d*. The labels inside individual panels show the mean synaptic weight ⟨*w*⟩. Parameters: *A* = 2.5, *f*_CR_ = 10 Hz and *σ* = *d*/4 Π. Raster plots in panels **(A,E,I, M)** are shown for *s* = 0.08*L*. Connectivity diagrams **(F–H,J–L, N–P)** were recorded at the end of the 1000 s stimulation period. Raster plots **(E,I,M)** show the last second of this period.

**FIGURE 6:**
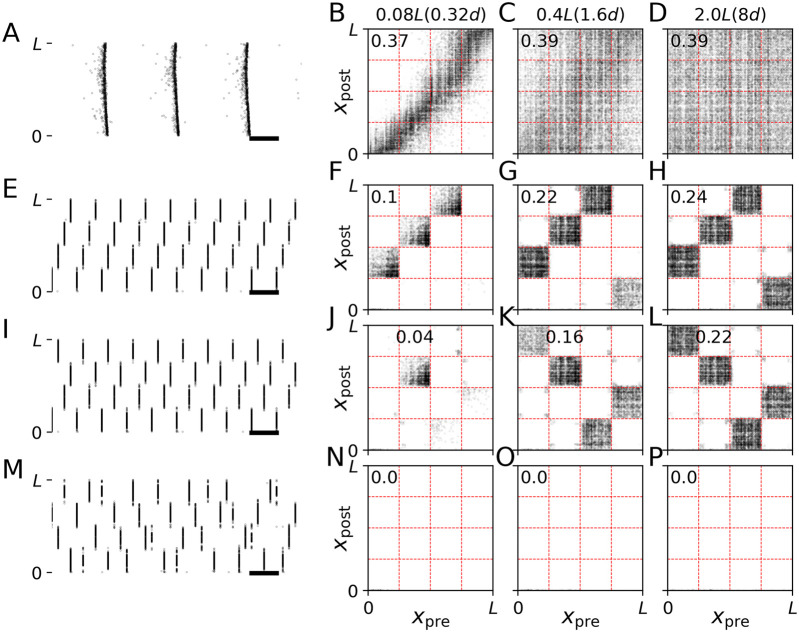
Synaptic pathways induced by CR stimulation with single-pulse stimuli. Results of stimulation of the LIF network model prior to stimulation **(A–D)**, and during simulation with non-shuffled CR with CR sequences “I,II,III,IV” **(E–H)** and “I,IV,II,III” **(I–L)** and shuffled CR **(M–P)** are shown. In contrast to Figure 5, we used single-pulse stimuli. The raster plots on the left show neuronal spiking activity. Black horizontal bars mark a time interval of 100 ms. The other panels show connectivity diagrams with dark colors marking strong connections and light gray marking weak connections between presynaptic neurons at locations *x*_pre_ and postsynaptic neurons at locations *x*_post_. Different columns show results for networks with different synaptic length scales, *s* (see labels at the top of respective columns). Labels inside the panels show the value of the mean synaptic weight. Parameters: *A* = 2.5 and *σ* = *d*/4 Π. Raster plots in panels **(A,E,I, M)** are shown for *s* = 0.08*L*. Connectivity diagrams were recorded at the end of the 1000 s stimulation period. The raster plots on the left show the last second before stimulation onset **(A)** and the last second of the 1000 s of CR stimulation period **(E,I,M)**.

For simulations of CR stimulation, we used the networks for an initial mean synaptic weight of 0.45 from Figure 2 at *t* = 5000 s and started stimulation. Thus, the time point at *t* = 5000 s from Figure 2 corresponds to the onset of stimulation at *t* = 0 s in Figures 4–6, 8, 9.

**FIGURE 7:**
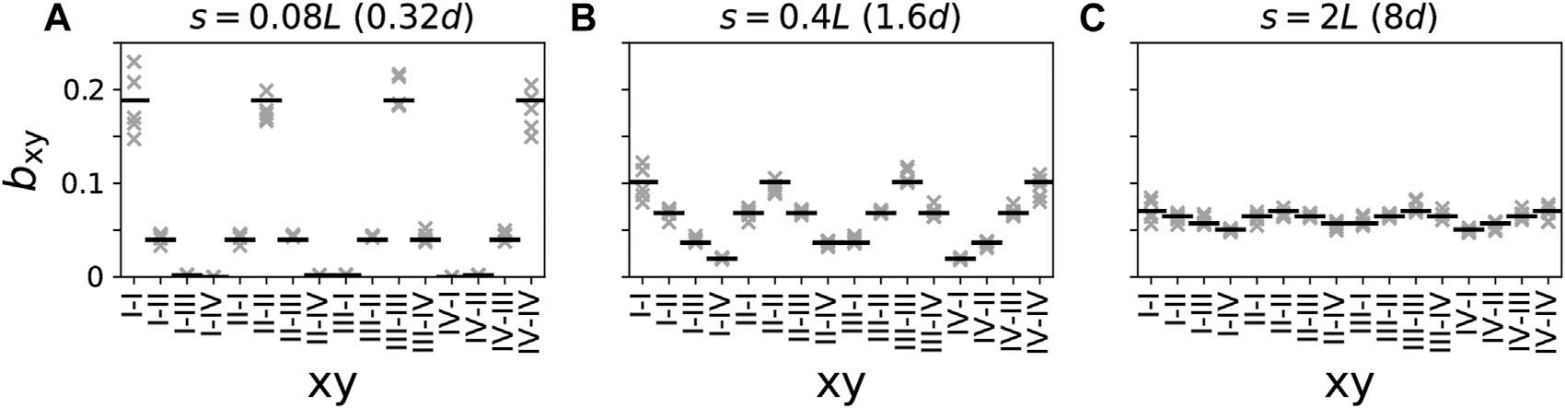
Relative numbers of connections between neuronal subpopulations closest to different combinations of stimulation sites for different synaptic length scales **(A–C)**. Black horizontal lines represents analytical estimates (Eq. 10) and crosses show data from five network realizations. Presynaptic and postsynaptic neuronal subpopulations are denoted by the Roman numeral of the closest stimulation site, i.e., “I-II” refers to the presynaptic subpopulation of all neurons within distance *d*/2 of stimulation site “I” and postsynaptic subpopulation of all neurons within distance *d*/2 of stimulation site “II”.

**FIGURE 8:**
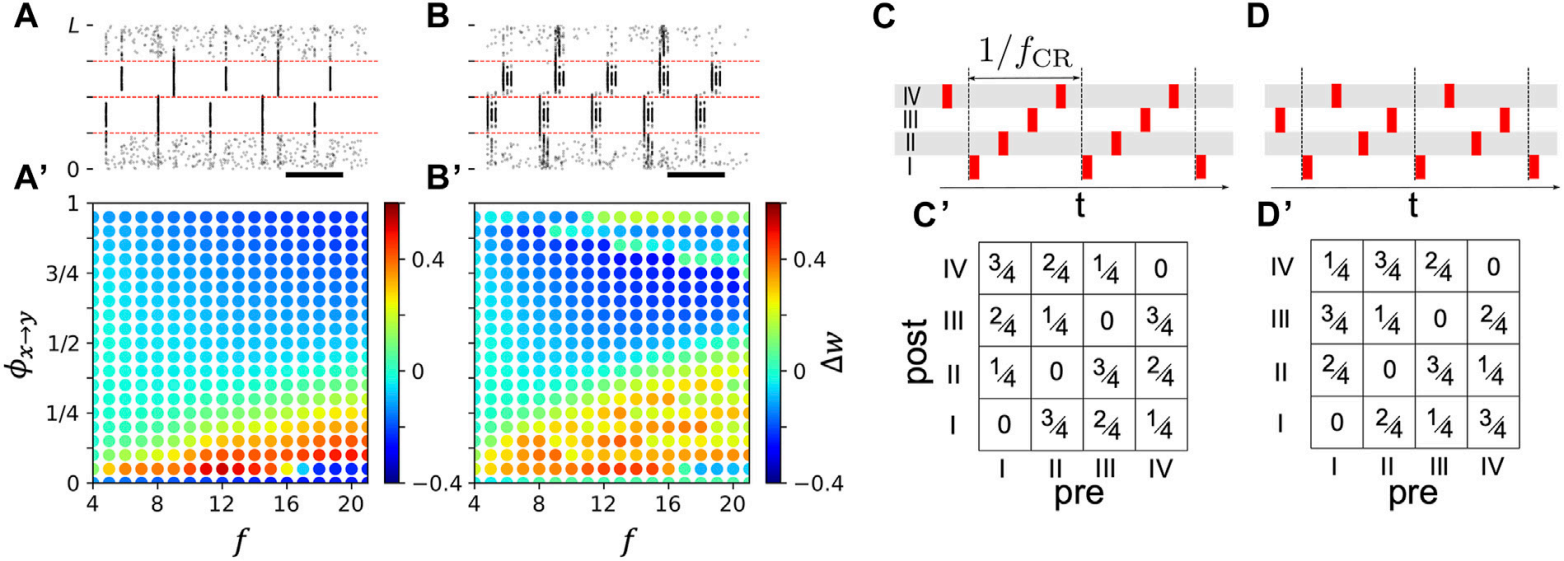
Stimulation-induced change of mean synaptic weight depends on phase lag between stimuli delivered to postsynaptic and presynaptic neuronal subpopulation. **(A)**: Raster plot of neuronal spiking activity during two-site stimulation with single-pulse stimuli for *ϕ*_*x*→*y*_ = 0.3. Phase lags were normalized to one, i.e., *ϕ*_*x*→*y*_ ∈ [0, 1), such that *ϕ*_*x*→*y*_/*f* is the time lag between the onsets of stimuli delivered to population *y* and *x*, respectively. **(A)**’: Change of mean synaptic weight of synapses between postsynaptic neurons closest to a site located at 5*L*/8 and presynaptic neurons closest to a site located at 3*L*/8 in the first 20 s of stimulation (enclosed by red dashed lines in **(A)** as function of *ϕ*_*x*→*y*_ and stimulation frequency, *f*. **(B)** and **(B)**’: Same as **(A)** and **(A′)** but for stimulation with burst stimuli with three pulses per burst and an intraburst frequency of 130 Hz. **(C, D)**: Examples of CR sequences (top). Roman numerals denote stimulation sites. Bottom panels show corresponding matrices of resulting phase lags between stimuli delivered to postsynaptic and presynaptic neurons affected by respective stimulation sites.

**FIGURE 9:**
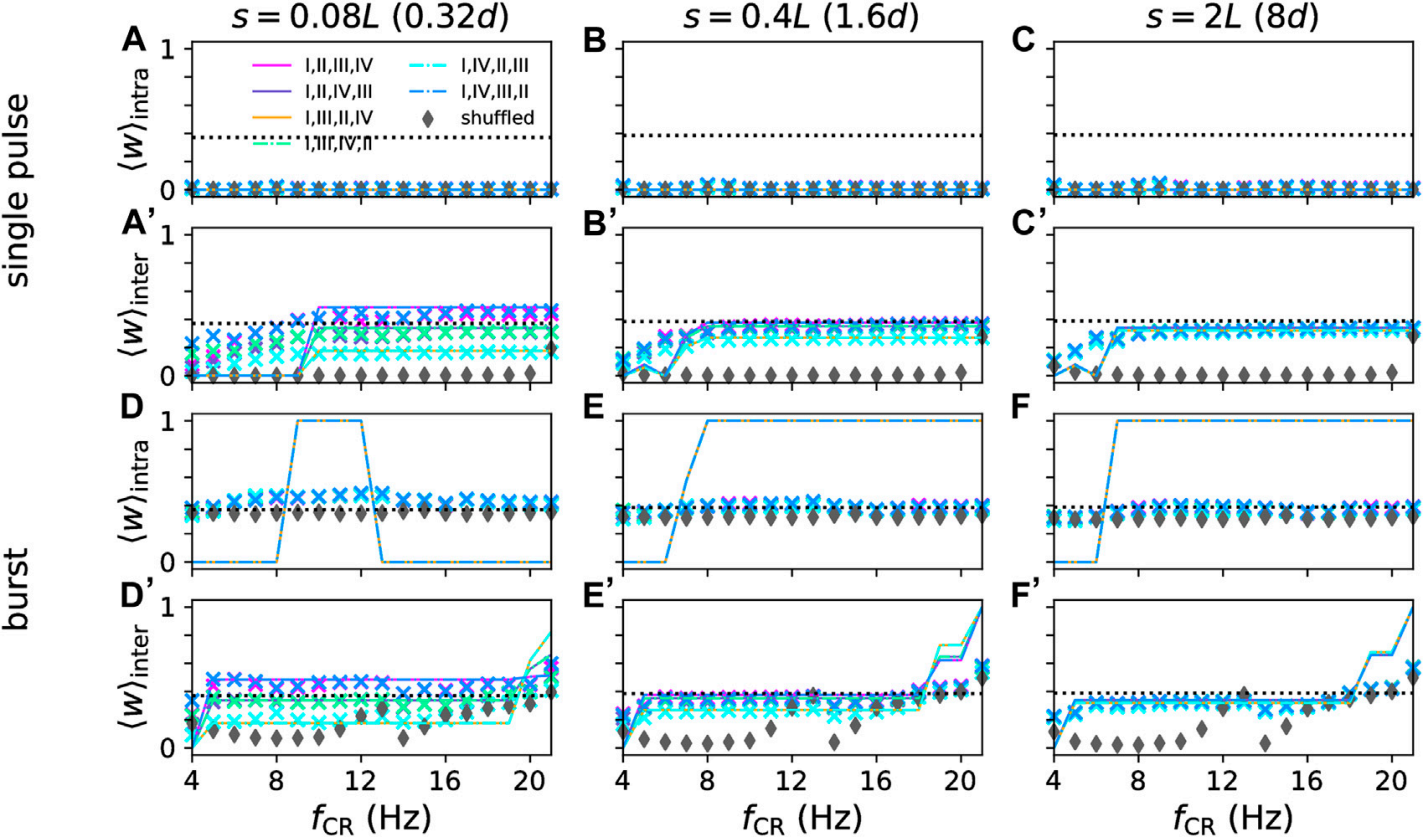
Mean synaptic weights of intra- **(A–C)** and **(D–F)** and inter-population synapses **(A’–C’)** and **(D’–F’)** after long stimulation with non-shuffled CR with different CR sequences and shuffled CR. Approximations from Eq. 14 (lines) for *t* = 3000 s are compared to simulation results after 3000 s of stimulation (symbols) for networks with different synaptic length scales, *s*, (columns) and for single-pulse **(A–C)** and **(A’–C’)** and burst stimuli **(D–F)** and **(D'-F')**. Colors mark different CR patterns (see legend in panel **(A)** and Figure 4). Parameters: *A*_stim_ = 2.5, *σ* = *d*/4 Π.

The relative numbers of connections *b*_*xy*_ in Figure 7 were obtained by averaging over five network realizations. The same networks were used in the simulations for Figure 8. Analytical approximations in Figure 7 and for the approximations in Figure 9 were obtained from Eq. 10.

Estimates of the mean weight change during stimulation, Δ*w*, in Figures 8A’, 8B’, were obtained by averaging the weights of all synapses between presynaptic neurons within a distance of less than *d*/2 from the stimulation site at *x*_II_ = 3*L*/8 and postsynaptic neurons within a distance of less than *d*/2 from the stimulation site at *x*_III_ = 5*L*/8, at the onset of stimulation and after 20 s of stimulation with a phase lag *ϕ*_*x*→*y*_ and a stimulation frequency *f*. Then the corresponding mean synaptic weights were averaged over five network realizations.

## 3 Results

We performed simulations of non-shuffled and shuffled CR stimulation of networks of LIF neurons with STDP. Before stimulation, the networks were in a strongly connected, synchronized state. Specifically, we used the networks obtained for an initial mean synaptic weight of 0.45 from Figure 2 after a simulation time of 5000 s. We delivered CR stimulation with the primary goal to downregulate synaptic weights and drive the network into the attractor of a stable, weakly connected desynchronized state (see Figure 2) such that stimulation entailed long-lasting desynchronization effects (see Figure 4). We recorded traces of the mean synaptic weight ⟨*w*(*t*)⟩ and the Kuramoto order parameter, *ρ*(*t*), quantifying the degree of neuronal synchrony (Eq. 7).

### 3.1 CR stimulation-induced dynamics depends on network structure

Representative trajectories of the Kuramoto order parameter, *ρ*(*t*), and the mean synaptic weight, ⟨*w*(*t*)⟩, before, during, and after CR stimulation with burst stimuli are shown in Figure 4. Simulations were performed for networks with different synaptic length scales. CR stimulation induced complex dynamics of the Kuramoto order parameter and the mean synaptic weight (Figure 4). While various CR patterns led to acute desynchronization (reflected by low values of Kuramoto order parameter during stimulation), the degree of long-term synchronization varied across networks with different synaptic length scales and stimulation parameters. In networks with short synaptic length scales, *s*, non-shuffled CR with different CR sequences led to different values of the mean synaptic weight during CR stimulation. In contrast, non-shuffled CR stimulation of networks with long synaptic length scales led to similar mean synaptic weights during stimulation (compare first and last column in Figure 4).

After cessation of stimulation, the networks freely evolved and approached either the synchronized, a desynchronized, or a partially synchronized state. In the latter, neurons in the immediate vicinity of stimulation sites remained synchronized whereas neurons further away from stimulation sites showed desynchronized activity (see raster plots of spiking activity and snapshots of synaptic connectivity for simulations of shuffled CR with high CR frequencies in Supplementary Figure S1). In networks with short synaptic length scales, it took a long time for the networks to approach a stationary state with transient dynamics occurring on time scales that were even longer than the stimulation period (Figure 4, left column). In some cases, non-shuffled CR with different sequences also led to different long-term effects of stimulation (Figure 4F). Surprisingly, in these cases, different stationary states were approached even though similar mean synaptic weights were obtained at the end of the stimulation period, indicating a complex attractor landscape of the stationary states.

For most parameter sets used in Figure 4 and sufficiently long stimulation periods, shuffled CR led to a more pronounced reduction of the mean synaptic weight during stimulation and lower values of the Kuramoto order parameter after cessation of stimulation than non-shuffled CR. For large CR frequencies, only shuffled CR kept the system from returning to the synchronized state. Here, the system approached a partially synchronized state that persisted even for long simulation times (we simulated up to 10000 s after cessation of stimulation) (Figures 4G–I). However, at the beginning of the stimulation period, non-shuffled CR led to a faster reduction of the mean synaptic weight than shuffled CR for a low stimulation frequency (see Figures 4A–C). It is currently unclear whether this effect is strong enough to enable long-lasting desynchronization effects after non-shuffled CR for shorter stimulation periods than required for long-lasting desynchronization after shuffled CR.

### 3.2 CR pattern determined spatial orientation of stimulation-induced synaptic pathways

Next, we analyzed the effect of non-shuffled CR on the synaptic weight dynamics. Snapshots of connectivity diagrams during CR stimulation are shown in Figure 5. Non-shuffled CR upregulated certain populations of synaptic connections while down-regulating others, thereby inducing strongly connected clusters of neurons. To analyze which synapses were affected, we drew red dashed lines in the connectivity diagrams in Figure 5 at coordinates *L*/4, *L*/2, and 3*L*/4, i.e., marking halfway points between adjacent stimulation sites. Vertical lines separate presynaptic neuron coordinates into four groups, each containing presynaptic neurons that were the closest to the same stimulation site. Accordingly, horizontal lines separate postsynaptic neuron coordinates into four groups containing postsynaptic neurons that were the closest to the same stimulation site. As can be seen in Figure 5, these lines approximately separate synaptic connections into different populations with presynaptic and postsynaptic neurons closest to the same stimulation sites.

Remarkably, non-shuffled CR with different CR sequences up- and downregulated different synaptic populations, thereby shaping the orientation of groups of strong synapses marking pathways of strong synaptic connections in the network. For example, in Figure 5H, the CR sequence I,II,III, IV induced the following pathways of strong synaptic connections I → II, II → III, III → IV, IV → I. Here, X → Y, denotes strong synaptic connections between presynaptic neurons with closest site X and postsynaptic neurons with closest site Y. In contrast, the CR sequence I, IV, II, III induced the pathways I → IV, II → III, III → I, and VI → II of strong synapses (Figure 5L).

In networks with short synaptic length scales, some of these pathways only contained a small number of synapses (compare Figures 5F–H; Figures 5J–L). This resulted in a different structure of CR stimulation-induced synaptic pathways due to the lag of synaptic connections between certain neuronal subpopulations. Consequently, different CR sequences led to different mean synaptic weights after non-shuffled CR stimulation of networks with short synaptic length scales if up- and downregulated blocks contained different numbers of synaptic connections.

Comparing the results for non-shuffled CR using burst stimuli (Figure 5) as opposed to single-pulse stimuli (Figure 6), we found that both induced similar patterns of strongly connected synaptic pathways. Noteworthy, for both types of stimuli, no such pathways were induced by shuffled CR variants (Figures 5, 6, bottom row). This was robust with respect to different realizations of random CR sequences during shuffled CR. These results suggests that the structure of CR stimulation-induced synaptic pathways is mainly determined by the CR pattern and less affected by the employed type of stimulus.

### 3.3 Stimulus shape determines network structure in the vicinity of stimulation sites

Next, we studied how stimuli affected the local network structure in the vicinity of the stimulation sites. In the connectivity diagrams in Figures 5, 6, the local network structure consists of the populations of synapses that are located along the diagonals. We will refer to these synapses as intra-population synapses as they connect presynaptic and postsynaptic neurons that are close to the same stimulation site, as opposed to inter-population synapses where presynaptic and postsynaptic neurons are close to different sites. We thereby follow the terminology of previous studies in which a rectangular spatial stimulus profile was implemented (i.e., all neurons within a certain distance of a stimulation site received the same stimulation current (Kromer et al., 2020; Kromer and Tass, 2022)). Of note, in the present study, the stimulation current decays with increasing distance from the stimulation site (Equation 8).

Stimulation-induced reshaping of intra-population synapses was strongly affected by the employed stimulus shape. Delivering CR stimulation with burst stimuli and a spatial stimulus profile (Eq. 8) caused a reshaping of local synaptic connectivity. Specifically, synaptic connections with postsynaptic neurons that were close to the stimulation site were upregulated. In contrast, connections with postsynaptic neurons that were further away from their closest stimulation site were downregulated. This resulted in the horizontal black regions along the diagonals in the connectivity diagrams during CR stimulation with burst stimuli in Figure 5. This complex local network structure was in marked contrast to CR stimulation with single-pulse stimuli where all intra-population synapses were downregulated (Figure 6). Consequently, in networks with short synaptic length scales, a substantial reduction of the mean synaptic weight can be achieved if the “right” stimulus is used, i.e., single pulse instead of burst stimuli, in our model. Note that inter-population synapses were affected in a similar way for both burst and single-pulse stimuli (Figures 5, 6), as discussed in the previous paragraph.

### 3.4 Effect of stimulus shape and CR pattern on synaptic weights

The results in Figure 7 show that networks with short synaptic length scales (Figure 7A) contain mostly intra-population synapses; therefore, for effective synaptic downregulation, the focus should be on reducing the strength of intra-population synapses. In contrast, in networks with long synaptic length scales (Figure 7C), the distribution of synapses across the different combinations of presynaptic and postsynaptic neuronal subpopulation is close to uniform. Thus, the major portion of synapses are inter-population synapses, and the focus should be on reducing the strength of such synapses when aiming for synaptic downregulation.

Combining the pronounced difference between the distributions of synapses in networks with different synaptic length scales and the results on the different effects of the CR pattern and the stimulus shape on the connectivity diagrams in Figures 5, 6 from the previous sections, we formulate the hypothesis, that selecting an effective stimulus shape can substantially improve stimulation-induced synaptic weight reduction in networks with short synaptic length scales; whereas, in networks with long synaptic length scales the selection of the CR pattern strongly affects synaptic weight reduction. Here, we use the term CR pattern to refer to the spatiotemporal characteristics of stimulus deliveries, characterized by the CR sequence, the presence or absence of shuffling as well as the CR frequency. In contrast, the stimulus shape is characterized by the stimulation amplitude, the number of stimulus pulses per burst, and the intra-burst frequency (Figure 3).

Accordingly, comparing the results for networks with short synaptic length scales in Figures 5, 6, we see that the difference between mean synaptic weights after CR stimulation with single-pulse (Figure 6) and burst stimuli (Figure 5) is substantial for all three CR patterns, i.e., for non-shuffled CR with sequence I,II,III, IV compare Figures 5F, 6F and with sequence I,IV,II, III compare Figures 5J, 6J, and for shuffled CR compare Figures 5N, 6N. In contrast, for long synaptic length scales the difference between connectivity diagrams and mean synaptic weights for the same CR pattern for burst and single pulse stimuli is smaller (compare Figures 5H, 6H, 5L, 6L; Figures 5P, 6P, respectively).

### 3.5 CR pattern selection for STDP-induced synaptic weight reduction

Finally, we studied how synaptic weight reduction depended on the selected CR pattern. We focused on the case where neuronal responses to stimuli were short compared to the inter-stimulus intervals.

The dynamics of the mean synaptic weight for a single synaptic population ⟨*w*_*x*→*y*_⟩ during CR stimulation strongly depends on the mean rate of weight change (Eq. 11 for non-shuffled CR). Note that for shuffled CR, the dynamics is more complex and was studied for single-pulse stimuli in Kromer et al. (2020).

For non-shuffled CR, an estimate of the function *S* (Eq. 11) for given CR parameters can be obtained by stimulating two subpopulations with stimulation frequency *f* for a time period *T* (Kromer and Tass, 2022) and evaluating the change of the mean synaptic weight of synapses interconnecting the two subpopulations Δ*w* = ⟨*w*_*x*→*y*_(*t*_0_ + *T*)⟩ − ⟨*w*_*x*→*y*_(*t*_0_)⟩. This setup is illustrated in Figures 8A,B for single-pulse and burst stimuli, respectively.

In Figures 8A’, 8B’, we show Δ*w* for single-pulse stimuli (Figures 8A, A’) and burst stimuli (Figures 8B, B’) with three pulses per burst for different stimulation frequencies *f* and phase lags, *ϕ*_*x*→*y*_, between stimuli delivered to the two subpopulations. We used short stimulation periods of *T* = 20 s to minimize the effect of the hard bounds on individual synaptic weights. Note that for long stimulation times *T* → *∞*, synaptic weights approach the hard bounds, and Δ*w* either approaches −⟨*w*(*t*_0_)⟩ indicating a reduction of individual weights towards the lower hard bound, or 1 − ⟨*w*(*t*_0_)⟩ indicating an increase of individual weights towards the upper hard bound.

In a wide range of stimulation frequencies, the sign of Δ*w* depended on the phase lag *ϕ*_*x*→*y*_, indicating that the adjustment of the phase lag between stimuli determines whether synapses between these neuronal subpopulations strengthen (Δ*w* > 0) or weaken (Δ*w* < 0).

In the schematics in Figures 8C, C’, and Figures 8D, D’, we show the phase lags between stimuli delivered to different stimulation sites for two CR sequences. For *M* = 4 stimulation sites, each CR sequence induces *M* times the phase lags 0, 1/*M*, 2/*M*, 3/*M*, … , (*M* − 1)/*M*, between neuronal subpopulations that are closest to the *M* stimulation sites; however, the pairs of neuronal subpopulations that receive stimuli at the phase lags vary across CR sequences (Figures 8C’, D’). As a consequence, the resulting change of the mean synaptic weight (Eq. 13) may depend on the CR sequence in inhomogeneous networks $b_{x_{1}y_{1}}\neq b_{x_{2}y_{2}}$ . For a given CR sequence, the mean synaptic weight at time *t* > *t*_0_ during stimulation can be approximated by

$${\left.\left\langle w\left(t\right)\right\rangle \right|}_{\phi }\approx \underset{xy}{\sum }b_{xy}{\left[\left\langle w_{x\to y}\left(t_{0}\right)\right\rangle +S\left(J\left(\phi_{x\to y},f_{\text{CR}}\right),\left\langle w_{x\to y}\left(t_{0}\right)\right\rangle \right)t-t_{0}\right]}_{\text{clip},0,1}.$$ (14)

***ϕ*** denotes the matrix of the *M* ^2^ phase lags *ϕ*_*x*→*y*_ between the different combinations of postsynaptic subpopulations, *y*, and presynaptic subpopulations, *x* (see examples in Figures 8C’, D’).

Approximating $S(J\left(\phi_{x\to y},f_{\text{CR}}\right),\left\langle w_{x\to y}\left(t_{0}\right)\right\rangle )t-t_{0}\approx \Delta w(\phi_{x\to y},f=f_{\text{CR}})(t-t_{0})/T$ and *b*_*xy*_ using Eq. 10, we evaluated ${\left.\left\langle w\left(t\right)\right\rangle \right|}_{\phi }$ for the matrices ***ϕ*** for different CR sequences and networks with different synaptic length scales. Note that for long stimulation times *t* − *t*_0_ → *∞* the sign of Δ*w* is sufficient as the mean weight of synapses between subpopulations will either approach zero or one. The results are compared to simulations of the LIF network in Figure 9.

As can be seen in Figure 9, the approximations based on Eq. 14 well approximated the mean weight of inter-population synapses for single-pulse and burst stimuli (Figures 9A’–F’) and for intra-population synapses when single-pulse stimuli were used (Figures 9A–C). For burst stimuli, large deviations between approximations and simulation results occurred. These deviations were caused by the local network structure (see Figure 5), which was not considered by the approximations. Instead, approximations were solely derived based on the mean weight change for the synaptic populations and assumed that weights of all synapses within the population approach either one or zero. Furthermore, for low CR frequencies, neurons have enough time between stimuli to respond to input from other subpopulations, which causes deviations from the two-site stimulation results (Figure 8) used to calculate the approximations.

Overall, the stimulation-induced mean synaptic weight of intra-population synapses was more robust with respect to changes in the CR frequency than the one of inter-population synapses.

## 4 Discussion

CR DBS induced long-lasting therapeutic effects in Parkinsonian monkeys (Tass et al., 2012; Wang et al., 2016; Bore et al., 2022; Wang et al., 2022) and PD patients (Adamchic et al., 2014). Therapeutic long-lasting effects were also observed in PD patients who received non-invasive vibrotactile CR finger-tip stimulation (Pfeifer et al., 2021). Computational studies in plastic neural networks and networks of phase oscillators predicted that CR stimulation may have long-lasting effects resulting from a downregulation of plastic synaptic connections, in this way driving the network from a pathological state, prior to stimulation, into the basin of attraction of a stable physiological state in which the network remains after cessation of stimulation (Tass and Majtanik, 2006). In previous computational studies, CR stimulation was studied in different network models with plasticity, including networks of phase oscillators (Tass and Majtanik, 2006) and spiking neuronal networks (Popovych and Tass, 2012; Manos et al., 2018; Kromer et al., 2020). There, the focus was on the influence of stimulation parameters such as the CR frequency and the stimulus amplitude on synaptic downregulation and long-lasting desynchronization effects. Here, we focused on networks with spatially dependent synaptic connections and derived stimulation strategies for CR stimulation that harness spatially inhomogeneous network structure for efficient synaptic downregulation during stimulation.

Multiple stable states can coexist in plastic networks (Seliger et al., 2002; Zanette and Mikhailov, 2004; Maistrenko et al., 2007; Madadi Asl et al., 2016; Madadi Asl et al., 2018a; Madadi Asl et al., 2018b). The networks studied here showed coexisting synchronized and desynchronized stationary states (Figure 2) and partially synchronized states in which the system remained for several hours after cessation of shuffled CR stimulation with burst stimuli (Figure 4 and Supplementary Figure S1). During CR stimulation, we observed rapid changes of the synaptic connectivity that were followed by sometimes long and complex transient dynamics after cessation of stimulation before the network eventually approached either stationary state (Figure 4). These transient dynamics included short periods of rebound synchronization (Figures 4D–I) (Tass and Hauptmann, 2006) and long transient desynchronization that was sometimes followed by a relaxation back to a stationary synchronized state (Figure 4F). These results suggest that in a clinical setting therapeutic CR stimulation may be followed by epochs of transient dynamics, that are potentially longer than the actual stimulation period.

Figure 4 also tells us that pronounced acute desynchronization by CR stimulation does not necessarily imply long-term desynchronization: For instance, while shuffled CR as well as all types of non-shuffled CR induced a pronounced acute desynchronization for *f*_CR_ = 21 Hz, shuffled CR entailed partially synchronized network dynamics and the system resynchronized after non-shuffled CR stimulation (see Figures 4G–I). Hence, in this computational model, acute desynchronization, measured during stimulus delivery, does not provide sufficient information for calibration and/or prediction of long-term outcome of CR stimulation. In clinical applications, this may make it difficult to predict long-term therapeutic outcome based on solely measuring acute effects during stimulus delivery.

Targeting synaptic plasticity for long-lasting therapeutic effects remains challenging as stimulation solely provides control over the synaptic weight dynamics during stimulation. However, even networks with similar mean synaptic weights at the end of the stimulation period may approach different stationary states after cessation of stimulation (see, e.g., Figure 4F). Furthermore, it is currently not possible to assess synaptic weights directly during stimulation. In the present study, we implemented a procedure suggested by Kromer and Tass (2022) during which an estimate of the synaptic weight dynamics induced by the employed stimulation is obtained during a two-site stimulation setup (Figure 8), and then this estimate is used to predict which CR patterns lead to the desired synaptic weight reshaping (Figure 9). However, it is unclear how direct estimates of synaptic weight changes can be obtained in patients as classic STDP experiments, e.g., as performed in Bi and Poo (1998), are not feasible. For such a procedure, one may rely on results from corresponding animal experiments or indirect measures, such as changes of evoked responses following stimulation as commonly used in the context of plasticity induced by transcranial magnetic stimulation (Kozyrev et al., 2018; Schwab et al., 2019).

Our results on CR stimulation of neuronal networks with spatially dependent synaptic connections and STDP revealed that fundamental characteristics of such networks can be harnessed to adjust CR stimulation for efficient synaptic weight downregulation. Considering CR stimulation of networks with different synaptic length scales, our results suggest that the ratio between the characteristic distance between stimulation sites, *d*, and the characteristic synaptic length scale, *s*, determines whether a network is dominated by intra-population connections (*s* ≪ *d*) or inter-population synapses (*s* ≫ *d*). In the former case, the shape of the employed stimulus, characterized by parameters such as the number of pulses per stimulus burst, the intra-burst frequency, and the stimulus amplitude, is critical for efficient synaptic weight reduction. Our computational results suggested that such a stimulus could be combined with the shuffled CR pattern (often referred to as CR with rapidly varying sequence, Tass and Majtanik (2006)) for effective synaptic downregulation. We find that shuffled CR is capable of down-regulating synaptic connections between separately stimulated neuronal subpopulation, whereas some of these connections remain strong after non-shuffled CR (Figures 5, 6). The results in Figure 4 further suggest that long-lasting desynchronization effects of shuffled CR are more robust with respect to changes of the CR frequency and across networks with different synaptic length scales than those of non-shuffled CR. This is in accordance with previous computational (Kromer and Tass, 2024) and experimental results in Parkinsonian monkeys that reported that shuffled CR outperformed non-shuffled CR in terms of long-lasting therapeutic effects (Wang et al., 2022). We speculate that in some target brain regions, it may not be necessary to stimulate according to a CR pattern to induce long-lasting therapeutic effects as long as appropriate stimuli are employed. In this context, we note that periodic delivery of burst stimuli to the external segment of the globus pallidus, which is strongly interconnected with the STN, of dopamine-depleted mice induced long-lasting therapeutic effects that depended on the stimulus triggering certain neuronal responses (Spix et al., 2021). In contrast, if networks consist of mostly inter-population synapses, our results suggest that the employed CR pattern is of great importance, and that the choice of parameters characterizing the CR pattern, such as the CR frequency and the CR sequences and their shuffling, has a strong impact on synaptic weight reduction, as suggested by our computational results (Figures 5, 6). So far, clinical studies in PD patients used CR frequencies of 3–20 Hz that were adjusted to the individual patients’ dominant peaks in the LFP power spectrum between 2 and 35 Hz (Adamchic et al., 2014). An adjustment of the CR frequency to the frequency of the dominant synchronous rhythm was suggested by the original computational studies on CR in networks of phase oscillators (Tass, 2003b). Later, computational studies in plastic networks found a non-trivial dependence of long-lasting effects on the CR frequency and observed substantially weaker long-lasting effects for certain unfavorable frequencies (Manos et al., 2018; Kromer and Tass, 2020). Another study found that the interplay of synaptic transmission delays, plasticity parameters, and stimulation determines ranges of unfavorable CR frequencies. Robust long-lasting desynchronization was observed for low CR frequencies (approximately ≤20 Hz for four stimulation sites) (Kromer et al., 2020). As details on individual plasticity parameters in the STN are currently not known (see, however, Shen et al. (2003)), it remains to be seen to which extend the CR frequency impacts long-lasting effects in STN CR DBS.

We further found that carefully selected stimuli have the potential to induce non-trivial local network structure in the vicinity of the stimulation site (Figure 5). In particular, CR stimulation employing burst stimuli resulted in the strengthening of local synaptic connections with postsynaptic neurons that were very close to the site and a weakening of the connections with postsynaptic neurons that were further away from the site (Figure 5). This is a consequence of a complex interplay of synaptic plasticity and the distance dependence of stimulus-induced spiking responses due to the spatial stimulus profile (Eq. 8). Specifically, neurons close to the center of the stimulus profile experienced stronger stimulation (Eq. 8) that was able to induce one neuronal spike per stimulus pulse. In contrast, neurons further away from the center responded only reliably to the first stimulus pulse in each stimulus burst as they experienced a weaker stimulation that was not able to cause a spiking response shortly after a neuron’s spike. As a consequence, several postsynaptic spikes followed one presynaptic spike, leading to the upregulation of synapses with postsynaptic neurons close to the center of the stimulus profile (Figure 5). In marked contrast, single-pulse stimuli caused a single synchronized spiking response of high fidelity that is known to lead to synaptic downregulation in networks with axonal transmission delays that are longer than the width of the synchronized spiking response (Lubenov and Siapas, 2008; Knoblauch et al., 2012). Here, neurons were distributed along a line and the stimulus profile depended solely on the distance to the stimulation site. It would be interesting to study the stimulus-induced local network structures in higher-dimensional distributions of neurons with stimulus profiles without spherical symmetry, which could be realized by directional steering in DBS (Contarino et al., 2014) or as a consequence of anisotropic tissue in the target brain area (McIntyre et al., 2004).

It remains to be shown whether CR stimulation with single pulses (Figure 6) as opposed to bursts (Figure 5) is actually feasible in pre-clinical and clinical applications. So far, in pre-clinical and clinical CR studies bursts were used instead of single pulses (Tass et al., 2012; Adamchic et al., 2014; Wang et al., 2016; Bore et al., 2022; Wang et al., 2022). Based on the results obtained above, one may try to deliver single pulse CR as it might be more effective in inducing synaptic downregulation. The decoupling properties of single-pulse stimulation were also observed in hippocampus (Lubenov and Siapas, 2008); however, it is unclear whether this also applies to target regions for DBS in PD, e.g., the STN. We also want to note that qualitatively different model neurons as well as biological neurons may require stronger stimuli. In this case, bursts may provide stimuli of sufficient strength obeying tissue safety limits, as, e.g., demonstrated for tremor entrainment computationally as well as in a patient with spinocerebellar ataxia type 2 (SCA2) with periodic stimuli delivered through a subthalamic-thalamic DBS electrode (Barnikol et al., 2008). For the computational part of that study a network of FitzHugh-Nagumo neurons (FitzHugh, 1961; Nagumo et al., 1962) was used.

Our results suggest that the shuffling of CR sequences substantially improves the induced downregulation of synaptic weights (Figures 4, 9). While the outcomes of non-shuffled CR with different CR sequences differed in some networks, shuffled CR consistently led to either similar or better long-lasting desynchronization effects then non-shuffled CR with the best-performing sequence. It is important to note that this effect may be due to the considered spatial dependence of synaptic connections. In particular, the probability for synaptic connections between two neurons was decaying exponentially in our networks, which was motivated by previous works (Hellwig, 2000; Ebert et al., 2014). However, the resulting connectivity diagrams are approximately symmetric (Figure 1), and the networks do not realize a preferred direction of synaptic connections. The differences between CR sequences in networks with preferred direction of synaptic connections may be larger (see Kromer and Tass, 2024). For instance, based on Figures 8A’, D’, we would expect that the CR stimulation-induced mean synaptic weight for the CR sequence shown in Figure 8D would approach zero for long stimulation times if a network had only connections between neurons close to the following sites: I → III, II → IV, III → II, and IV → I, the first Roman numeral indicating the site closest to presynaptic neurons and the second one the site closest to postsynaptic neurons.

It will be important to test our results and predictions in detailed models of the basal ganglia region, in particular, the STN and globus pallidus. STN neurons are subject to several inputs from other nuclei, e.g., gamma-aminobutyric acidergic inputs from neurons in the external segment of the globus pallidus. In our model, we only considered excitatory neurons. Furthermore, synaptic inputs to the STN and its projections to other nuclei are somatotopically organized (Nambu, 2011). Studies in animal models of PD suggest that PD is accompanied by substantial synaptic reorganization (Fan et al., 2012; Miguelez et al., 2012; Chu et al., 2017; Pamukcu et al., 2020; Madadi Asl et al., 2022), and that the somatotopic organization of synaptic inputs is perturbed (Filion et al., 1988; Boraud et al., 2000; Cho et al., 2002). In our LIF model, stimulation-induced downregulation of synaptic connections leads to a stabilization of desynchronized neuronal activity. Evidence from other computational studies suggests that CR is not only able to downregulate synaptic connections, but also to restore more complex physiological connectivity (Hauptmann and Tass, 2010). This is important as more complex synaptic reshaping might accompany long-lasting therapeutic effects of CR stimulation in animal models and PD patients (Tass et al., 2012; Adamchic et al., 2014; Wang et al., 2016; Bore et al., 2022; Wang et al., 2022). The qualitatively different results for networks dominated by local as opposed to by non-local connections in our LIF model suggest that one might observed different stimulation-induced dynamics of connections between neurons representing similar *versus* different body parts. In the future, we plan on studying CR stimulation in more detailed computational models to understand how more complex synaptic connectivity affects long-lasting effects.

Our computational study reveals that knowledge of fundamental characteristics of target neuronal networks with spatially dependent synaptic connections and STDP can be harnessed to adjust CR stimulation parameters for improved synaptic downregulation. Downregulation of pathological synaptic connectivity (Tass and Majtanik, 2006) is currently assumed to be the mechanism by which CR stimulation induces long-lasting therapeutic effects in PD patients and related animal models (Tass et al., 2012; Adamchic et al., 2014; Wang et al., 2016; Pfeifer et al., 2021; Wang et al., 2022). Thereby, our results provide important hypotheses for future clinical studies and computational studies on CR stimulation of detailed computational models of target brain regions for deep brain stimulation, e.g., the STN, or vibrotactile stimulation, e.g., sensorimotor cortical areas.

## Data Availability

The original contributions presented in the study are included in the article/Supplementary material, further inquiries can be directed to the corresponding author. The code that was used to generate the figures in this article is available on github
