# Supplementary material for: Coordinated reset stimulation of plastic neural networks with spatially dependent synaptic connections
Source: Front Netw Physiol. 2024 May 28;4:1351815. doi: 10.3389/fnetp.2024.1351815 (PMC11165135; doi:10.3389/fnetp.2024.1351815)
Supplement: Supplementary file 1 [file DataSheet1.pdf]

Supplementary Material

Coordinated reset stimulation of plastic neural networks with spatially dependent synaptic connections

Justus A. Kromer*, Peter A. Tass

*** Correspondence:** Justus A. Kromer: jkromer@stanford.edu

1.1 Supplementary Figures

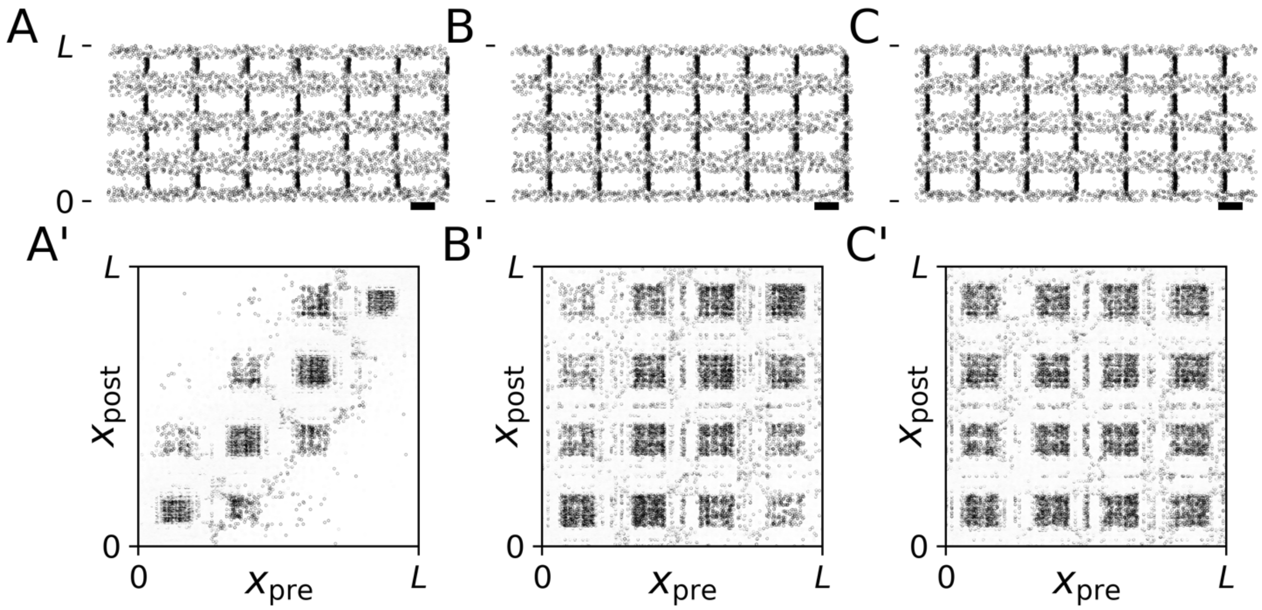

Supplementary Figure 1. Raster plots (A-C) and snapshots of connection diagrams (A'-C') for partially synchronized states 7000 sec after cessation of shuffled CR with stimulation frequency $f_{CR} = 21$ Hz. Corresponding trajectories of the Kuramoto order parameter and the mean synaptic weight are shown in Figures 4G-I in the main text. Columns show results for $s = 0.08\,L\ (0.32\,d)$ (A, A'), $0.4\,L\ (1.6\,d)$ (B, B'), and $2.0\,L\ (8\,d)$ (C, C'). The parameters are the same as the ones used in Figures 4G-I in the main text. The black horizontal bars in panels A-C mark 100 ms time intervals. Performing long simulations (up to 10000 sec) after cessation of stimulation, we found that the connectivity diagrams and raster plots did not change qualitatively indicating that the system remained in these partially synchronized states for a long time after shuffled CR with burst stimuli.
